# Postoperative day 1 serum cystatin C level predicts postoperative delayed graft function after kidney transplantation

**DOI:** 10.3389/fmed.2022.863962

**Published:** 2022-08-12

**Authors:** Yajuan Li, Bo Wang, Le Wang, Kewei Shi, Wangcheng Zhao, Sai Gao, Jiayu Chen, Chenguang Ding, Junkai Du, Wei Gao

**Affiliations:** ^1^Department of Anesthesiology and Center for Brain Science and Center for Translational Medicine, The First Affiliated Hospital of Xi’an Jiaotong University, Xi’an, China; ^2^Department of Anesthesiology, 521 Hospital of Norinco Group, Xi’an, China; ^3^Department of Renal Transplantation, The First Affiliated Hospital of Xi’an Jiaotong University, Xi’an, China; ^4^Department of Emergency, The First Affiliated Hospital of Xi’an Jiaotong University, Xi’an, China

**Keywords:** kidney transplantation, serum cystatin C, delayed graft function, least absolute shrinkage and selection operator, area under curve, clinical decision curve

## Abstract

**Background:**

Delayed graft function (DGF) commonly occurs after kidney transplantation, but no clinical predictors for guiding post-transplant management are available.

**Materials and methods:**

Data including demographics, surgery, anesthesia, postoperative day 1 serum cystatin C (S-CysC) level, kidney functions, and postoperative complications in 603 kidney transplant recipients who met the enrollment criteria from January 2017 to December 2018 were collected and analyzed to form the Intention-To-Treat (ITT) set. All perioperative data were screened using the least absolute shrinkage and selection operator. The discrimination, calibration, and clinical effectiveness of the predictor were verified with area under curve (AUC), calibration plot, clinical decision curve, and impact curve. The predictor was trained in Per-Protocol set, validated in the ITT set, and its stability was further tested in the bootstrap resample data.

**Result:**

Patients with DGF had significantly higher postoperative day 1 S-CysC level (4.2 ± 1.2 vs. 2.8 ± 0.9 mg/L; *P* < 0.001), serum creatinine level (821.1 ± 301.7 vs. 554.3 ± 223.2 μmol/L; *P* < 0.001) and dialysis postoperative (74 [82.2%] vs. 25 [5.9%]; *P* < 0.001) compared with patients without DGF. Among 41 potential predictors, S-CysC was the most effective in the parsimonious model, and its diagnostic cut-off value was 3.80 mg/L with the risk score (OR, 13.45; 95% CI, 8.02–22.57; *P* < 0.001). Its specificity and sensitivity indicated by AUC was 0.832 (95% CI, 0.779–0.884; *P* < 0.001) with well fit calibration. S-CysC yielded up to 50% of clinical benefit rate with 1:4 of cost/benefit ratio.

**Conclusion:**

The postoperative day 1 S-CysC level predicts DGF and may be used as a predictor of DGF but warrants further study.

## Introduction

Delayed graft function (DGF) is common after kidney transplantation. Once DGF occurs, 3.2 years graft survival decreases by 40%, 3 years death increases by 53%, and 3.5 years acute rejection increases by 38% (1–3). Current laboratory measurements, such as serum creatine (Scr), is inaccurate, and kidney graft biopsies are extremely invasive (4, 5). An early and non-invasive predictor of DGF is urgently needed to optimize timely postoperative clinical management. Pretransplant parameters have been analyzed with multivariate regressions for the formulation of predictive models that identify high-risk patients with DGF (6). The Irish model has an accuracy of 70% and has 16 clinical parameters of recipient- and donor-related factors, including cold ischemic time, donor terminal creatinine, donor body mass index, donation after cardiac death, and donor age (7). However, this model was built from the data of the United States Renal Data System in 2003 and does not meet the requirements of current clinical practice as marginal donor kidney grafts are widely used nowadays (8).

Scr, as the most used renal function biomarker, is used in the diagnosis of DGF, but it has low sensitivity when predicting DGF (9). Indeed, Scr may not rise before 50% loss of renal function and can be influenced by diet and muscle metabolism (10) and is the balance between creatinine production and excretion rather than a product of renal tubular injury (11). DGF in transplantation had been proved it was a specific manifestation of acute tubular necrosis (12). The products of renal tubular injury, such as kidney injury molecule-1 (KIM-1), interleukin-18 (IL-18), β-trace protein, and neutrophil gelatinase-associated lipocalin (NGAL), have promising diagnostic value for acute kidney injury or ischemic injury, but they have not been widely used clinically yet (13).

Previous studies reported Serum cystatin C (S-CysC) increased 24 h earlier than Scr after unilateral nephrectomy in kidney organ donors (14). S-CysC showed larger area under the curve than Scr in the prediction of postoperative renal dysfunction (0.73 vs 0.65; *P* = 0.01) (15). S-CysC is a 13.4 kDa cysteine protease inhibitor produced by nucleated cells at a constant rate and taken up by renal tubular epithelial cells without tubular secretion but is not re-absorbed into the circulation after being freely filtered by the glomeruli (16). Given that a significant increase in S-CysC level in the blood indicates tubular dysfunction, it has been used as a biomarker of glomerular filtration in chronic kidney disease (17–19). In prior study, the ROC curves showed that S-CysC had the largest AUC and the highest sensitivity and the highest diagnostic efficiency on postoperative day 1 after kidney transplantation (20). The first postoperative day S-CysC may be a potential predictor of DGF, but its clinical value has not been established and validated because of small sample size (18, 21). Therefore, we conducted this large-sample-size case control study to investigate the value of the first postoperative day S-CysC for predicting DGF.

## Materials and methods

### Study design

This study was approved by the ethics committee of the First Affiliated Hospital of Xi’an Jiaotong University (XJTUIAF2019LSL–008). This study was in accordance with the Transparent Reporting of a multivariable prediction model for Individual Prognosis Or Diagnosis. Written informed consent was waived because de-identified retrospective data were used. All the medical procedures adhered to the principles of the Declaration of Helsinki and the Istanbul Declaration, and all renal grafts were voluntarily donated. All organs (except kinship donor kidneys) were obtained by the Organ Procurement Organization of the First Affiliated Hospital of Xi’an Jiaotong University, supervised by the Red Cross Society of Shaanxi Province, and were allocated by China Organ Transplant Response System. Receptors were included in this cohort when they met the following criteria: (1) older than 18, (2) underwent kidney transplantation for end-stage kidney disease under general anesthesia, and (3) admitted to the First Affiliated Hospital of Xi’an Jiaotong University from January 1, 2017 to December 31, 2018. Donors who met any of the following criteria were excluded from the study: (1) combined kidney and other organ transplantation, (2) re-transplantation, and (3) missing clinical records of the S-CysC (24 h) or creatinine (72 h). All perioperative data (clinical symptoms, perioperative characteristics, postoperative kidney function examination, and postoperative complications) in kidney transplant patients from January 1, 2017 to December 31, 2018 were collected, cross-checked, and de-identified by a team of experienced clinicians. DGF was defined as post-transplant graft kidney dysfunction with no spontaneous 10% decline in serum creatinine in 72 h, and dialysis is required 72 h after transplantation (22). The data of included patients formed the Intention-To-Treat (ITT) set, which served as the validation set, and patients without missing values, formed the Per-Protocol set (PP), which served as the training set.

### Perioperative transplant procedures

The data of preoperative donors and recipients were obtained from the registry system of organ donation database and then evaluated and recorded in electronic medical record system by surgeons and anesthesiologists. Anesthesia management, surgery, and perioperative care followed standard institutional protocols. A triple immunosuppressive regimen with calcineurin inhibitors (CNIs), entericcoated mycophenolate sodium (EC-MPS; Myfortic, Novartis Pharma, Basel, Switzerland) and prednisone were treated all recipients. Cyclosporine A (CsA; Sandimmun Optoral, Novartis Pharma, Nuremberg, Germany) and tacrolimus (TAC; Prograf, Astellas Pharma, Deerfield, IL, United States) composed the CNIs. The initial dosages of CsA, TAC, EC-MPS and prednisone were 4.0–4.5 and 0.06–0.08 mg/kg/day, 1,080–1,440 and 10–20 mg/day, respectively. Rabbit anti-thymocyte globulin (rATG; thymoglobulin, Genzyme Ireland, Waterford, Ireland) at a dosage of 1.25–1.50 mg/kg/day as induction therapy during the surgery were given to all recipients in a total of 4–6 days after kidney transplantation.

### Donor and recipient characteristics

The collective data of all the recipients and donors were obtained and presented. For each patient, the baseline characters were screened: age, gender, body mass index, nationality, smoke, dialysis (hemodialysis, peritoneal dialysis, and hemodialysis vs. peritoneal dialysis), dialysis duration, comorbidities (hypertension, diabetes, coronary heart disease, cerebral infarction, phthisis, and hepatitis), pathogenesis of end-stage kidney disease (chronic glomerulonephritis, IgA nephropathy, and other kidney disease). The donor characteristics were as follows: donor (donation after cardiac death, donation after brain death, and kinsfolk), right or left kidney, and duration of ischemia (warm ischemia and cold ischemia). Operation factors, such as American Society of Anesthesiologist classification, iliac fossa, operation location, vascular anastomosis (internal iliac artery and arteria iliac externa), and time of operation. Intraoperative medication (propofol, dexmedetomidine, sevoflurane, sufentanil, remifentanil, and cisatracuramide) and intake and output volumes (crystal, colloid, intraoperative blood transfusion, intraoperative blood plasma, bleeding, and urine volume) were collected. Postoperative kidney function indexes, including S-CysC, Scr, glomerular filtration rate (GFR), urea nitrogen (BUN), and uric acid (UA) on the first day after surgery were compared.

### Statistical analyses

Between the DGF and non-DGF groups in the ITT set, the normally distributed continuous variables were presented as means ± standard deviations (SDs); otherwise, they were presented as medians (interquartile ranges). The categorical variables were reported as numbers (percentages). They were analyzed with independent-sample student’s *t*-tests, Mann–Whitney *U* test, Chi-square test, and Fisher’s exact test. All perioperative variables in the PP set were entered in the Least Absolute Shrinkage and Selection Operator (LASSO) selection process for the generation of a single predictive model of DGF. Missing predictor values in the ITT set (*N* = 517) were imputed through multiple imputation with chained equations. We used L1-penalized LASSO for multivariable analyses, augmented with 10-fold cross validation for internal validation. This logistic regression model penalizes the absolute size of the coefficients of a regression model to minimize the potential collinearity of variables measured from the same patient and model overfitting. The optimal diagnostic model and the most parsimonious model of LASSO regression were identified with minimum criteria and one standard error of the minimum criteria (the 1-SE criterion) in the 5 times multiple interpolation ITT sets. To compare the predictive effect of optimal model and the most parsimonious model in ITT set without multiple interpolation, the same items among the 5 optimal diagnostic models were collected by univariate analysis with *P* < 0.1, calculated their relative risk by multivariate analysis, and compared with the most parsimonious model by using the area under the receiver operating characteristic curve (AUC) and the DeLong method.

The internal validation of the single predictor was tested in the PP and ITT sets: (1) The predictive accuracy estimates and mean absolute error were calculated by 200 bootstrap resamples, (2) The calibration curves of the predictor on DGF were plotted and tested by Hosmer–Lemeshow test. (3) The optimal cut-off value of the single predictor was calculated by Youden’s index, and the relative risk was calculated by univariate logistic regression. (4) The clinical value of the predictor for DGF diagnosis was finalized through decision curve analysis (DCA). (5) A clinical impact plot was used in depicting the estimated number of high-risk patients and the true positive cases. All data were analyzed using R software (version 4.0.2) and Empower (X&Y Solutions, Inc., Boston, MA, United States). Packages in R that were used in this study were “rms,” “rmda” and “glmnet.” The reported statistical significance levels were two-side, with a *P* < 0.05 considered to be statistically significant.

## Results

### Development cohort

**FIGURE 1:**
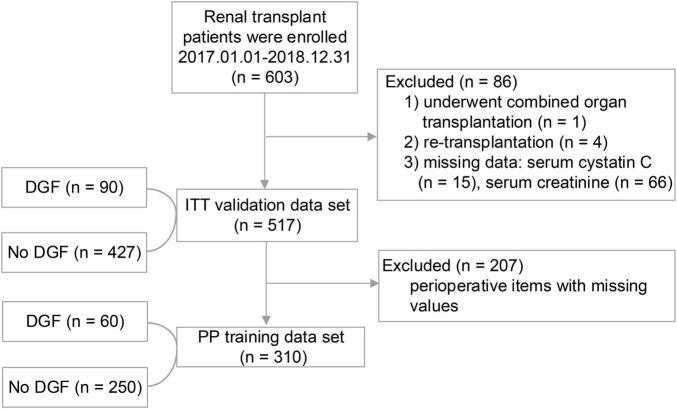
The study flowchart. During the study period, 603 patients underwent renal transplant surgery. A total of 86 (14.26%) patients were excluded because they did not satisfy the inclusion criteria. In the end, a total of 517 (Intention-To-Treat set) first time renal transplant recipients were included in the study, of whom 207 were excluded from the Per-Protocol analysis.

A total 603 kidney transplant patients received kidney transplants between January 1, 2017 and December 31, 2018. The ITT set had 517 patients, and 310 of these patients had missing data and formed the PP set (Figure 1). No significant difference in DGF incidence was found between the PP (19.35%) and ITT (17.41%). No significant differences in the demographic and clinical characteristics of the recipients were found between the DGF (*n* = 90) and non-DGF (*n* = 427) groups except for pneumonia (12 [2.8%] vs. 7 [7.8%]; *P* = 0.023; Table 1). The incidence rate of DGF in the different types of donors were 18.3%, 16.7% and 14.3%, respectively, corresponding to circulatory death, brain-death and living donation. The DGF group had a longer operation time, larger doses of propofol and remifentanil, and lower urine volume (*P* < 0.05; Table 2). The DGF group showed worse kidney function values (S-CysC, Scr, GFR, UA, and BUN) on the postoperative 1st day and longer length of hospital stay and progressed higher incidence of hospitalized complications, such as postoperative severe cardiovascular events (cardiac failure, arrhythmia, and acute coronary attack), pulmonary infection, gastrointestinal hemorrhage, acute rejection, renal artery stenosis, renal venous thrombosis, perirenal hemorrhage, and postoperative dialysis (Table 3).

**TABLE 1:** Demographics and clinical characteristics among patients in the development cohort who did or did not develop DGF.

Characteristic	Total	Non-DGF	DGF	*P*-value
Male, *n* (%)	372 (72.0%)	303 (71.0%)	69 (76.7%)	0.176
Age, mean (SD), y	35.9 (9.2)	35.6 (9.1)	37.7 (9.4)	0.050
BMI, mean (SD)	21.0 (3.0)	21.0 (3.0)	21.3 (3.2)	0.457
Smoke, *n* (%)	162 (31.3%)	131 (30.7%)	31 (34.4%)	0.484
**Nationality, *n* (%)**				0.805
Han Nationality	481 (93.0%)	396 (92.7%)	85 (94.4%)	
Hui Nationality	19 (3.7%)	16 (3.7%)	3 (3.3%)	
Other Nationality	17 (3.3%)	15 (3.5%)	2 (2.2%)	
**Dialysis, *n* (%)**				0.770
Hemodialysis	431 (83.5%)	358 (84.0%)	73 (81.1%)	
Peritoneal dialysis	57 (11.0%)	46 (10.8%)	11 (12.2%)	
Hemodialysis vs. peritoneal dialysis	28 (5.4%)	22 (5.2%)	6 (6.7%)	
Dialysis duration, median (IQR), m	15.0 (8.0–28.0)	15.0 (8.0–27.8)	14.0 (7.6–27.8)	0.644
**Comorbidities, *n* (%)**				
Hypertension	403 (78.0%)	328 (76.8%)	75 (83.3%)	0.175
Diabetes	15 (2.9%)	11 (2.6%)	4 (4.4%)	0.337
Coronary heart disease	21 (4.1%)	15 (3.5%)	6 (6.7%)	0.168
Cerebral infarction	19 (3.7%)	14 (3.3%)	5 (5.6%)	0.297
Pneumonia	19 (3.7%)	12 (2.8%)	7 (7.8%)	**0.023**
Hepatitis	36 (7.0%)	27 (6.3%)	9 (10.0%)	0.213
**Causes of ESRD, *n* (%)**				
Chronic glomerulonephritis	394 (76.2%)	324 (75.9%)	70 (77.8%)	0.701
IgA nephropathy	79 (15.3%)	65 (15.2%)	14 (15.6%)	0.936
Other kidney disease	57 (11.0%)	51 (11.9%)	6 (6.7%)	0.335

BMI, body mass index; ESRD, end stage renal disease.

**TABLE 2:** Surgical information is presented as mean (SD), median (IQR) or number (%).

Characteristic	Total	Non-DGF	DGF	*P*-value
**Donor, *n* (%)**				
DCD	180 (41.9%)	144 (40.3%)	36 (49.3%)	0.525
DBD	180 (41.9%)	152 (42.6%)	28 (38.4%)	0.884
Kinsfolk	70 (16.3%)	61 (17.1%)	9 (12.3%)	0.512
Kidney side, *n* (%)				0.912
Left	273 (52.8%)	226 (52.9%)	47 (52.2%)	
Right	244 (47.2%)	201 (47.1%)	43 (47.8%)	
**Duration of ischemia**				
Warm ischemia (min)	8.5 (4.8)	8.5 (4.8)	8.5 (4.5)	0.958
Cold ischemia (h)	5.9 (3.6)	5.9 (3.7)	5.7 (2.9)	0.663
**Notch location, *n* (%)**				0.620
Left iliac fossa	149 (28.8%)	125 (29.3%)	24 (26.7%)	
Right iliac fossa	368 (71.2%)	302 (70.7%)	66 (73.3%)	
**Vascular anastomosis, *n* (%)**				0.050
Internal iliac artery	166 (32.1%)	145 (34.0%)	21 (23.3%)	
Arteria iliac externa	351 (67.9%)	282 (66.0%)	69 (76.7%)	
ASA, *n* (%)				0.965
II	57 (11.0%)	47 (11.0)	10 (11.0%)	
III	293 (56.7%)	241 (56.4%)	52 (57.8)	
IV	167 (32.3%)	139 (32.6%)	28 (31.1%)	
**Intraoperative medication, mean (SD) or median (IQR)**				
Propofol, mg	1,412.3 (624.2)	1,381.4 (600.6)	1,558.6 (711.5)	0.014
Sufentanil, μg	30.6 (5.8)	30.6 (5.9)	30.4 (5.3)	0.765
Remifentanil, mg	2,203.1 (1,669.4–2,921.6)	2,161.2 (1,656.0–2,837.3)	2,468.3 (1,761.0–3,334.5)	**0.035**
Cisatracuramide, mg	23.8 (8.5)	23.5 (8.4)	25.3 (9.1)	0.067
Dexmedetomidine, μg	100.1 (70.0–149.7)	95.3 (70.0–146.6)	116.9 (72.0–155.8)	0.133
Sevoflurane, ml	4.9 (1.4)	4.9 (1.6)	5.0 (1.6)2	0.511
Operative Time, h	3.3 (0.7)	3.3 (0.7)	3.5 (0.8)	**0.029**
**Intraoperative volume infusion and loss, mean (SD) or median (IQR)**				
Crystal, ml	1,904.9 (546.3)	1,899.8 (563.5)	1,929.4 (457.8)	0.640
Colloid, ml	894.6 (340.4)	883.6 (330.5)	946.7 (381.7)	0.110
Red blood cells, *n*%	118 (22.9%)	100 (23.5%)	18 (20.0%)	0.476
Plasma, *n*%	49 (9.5%)	41 (9.6%)	8 (8.9%)	0.829
Bleeding, ml	150.0 (100.0–200.0)	150.0 (100.0–200.0)	150.0 (100.0–300.0)	0.678
Urine volume, ml	300.0 (150.0–500.0)	300.0 (200.0–500.0)	200.0 (100.0–300.0)	**<0.001**

DBD, donation after brain death; DCD, donation after cardiocirculatory death; ASA, American Standards Association. The P values in hold is P < 0.05.

**TABLE 3:** Renal function on the first day after surgery, postoperative complications while in hospital and length of stay.

Characteristic	Total	Non-DGF	DGF	*P*-value
**Kidney function**				
Serum cystatin C, mg/L	3.0 (1.1)	2.8 (0.9)	4.2 (1.2)	**< 0.001**
Serum UA, μmol/L	368.9 (94.0)	361.7 (91.9)	402.9 (97.3)	**< 0.001**
Serum BUN, mmol/L	18.1 (6.1)	17.4 (5.7)	21.6 (6.6)	**< 0.001**
Serum eGFR, ml/min/1.73m^2^	9.3 (6.7–13.3)	10.2 (7.3–14.8)	6.2 (4.8–9.0)	**< 0.001**
Serum SCR, μmol/L	600.8 (259.0)	554.3 (223.2)	821.1(301.7)	**< 0.001**
**Postoperative complications in hospital, *n*%**				
Cardiovascular events	24 (4.6%)	13 (3.0%)	11 (12.2%)	**< 0.001**
Pulmonary infection	54 (10.4%)	34 (8.0%)	20 (22.2%)	**< 0.001**
Gastrointestinal hemorrhage	5 (1.0%)	2 (0.5%)	3 (3.3%)	**0.012**
CRAD	2 (0.4%)	1 (0.2%)	1 (1.1%)	0.223
Renal infarction	2 (0.4%)	1 (0.2%)	1 (1.1%)	0.223
Acute rejection	9 (1.7%)	4 (0.9%)	5 (5.6%)	**0.002**
RAS	7 (1.4%)	1 (0.2%)	6 (6.7%)	**< 0.001**
RVT	15 (2.9%)	4 (0.9%)	11 (12.2%)	**< 0.001**
Perirenal infection	12 (2.3%)	10 (2.3%)	2 (2.2%)	0.945
Perirenal hemorrhage	6 (1.2%)	3 (0.7%)	3 (3.3%)	**0.034**
Urinary fistule	29 (5.6%)	23 (5.4%)	6 (6.7%)	0.631
Postoperative dialysis	41 (7.930%)	25 (5.855%)	16 (17.778%)	**< 0.001**
Length of stay, day	21.5 (9.3)	20.0 (7.3)	27.5 (11.8)	**< 0.001**

Data are presented as mean (SD), median (IQR) or number (%). Cardiovascular events are defined as postoperative cardiac failure, arrhythmia and acute coronary attack. GFR, glomerular filtration rate; SCR, serum creatinine; BUA, urea nitrogen; UA, uric acid; CRAD, chronic renal allograft dysfunction; RAS, renal artery stenosis; RVT, renal venous thrombosis. Postoperative dialysis: As an adverse event, during hospitalization after kidney transplantation, Incidence of dialysis 72 h after surgery. The P values in hold is P < 0.05.

### Predictor selection

**FIGURE 2:**
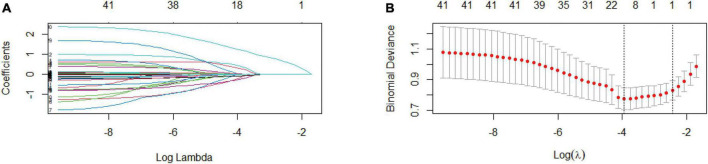
Exclude all missing values feature selection (Per-Protocol set) using the least absolute shrinkage and selection operator (LASSO) binary logistic regression model (*n* = 310). **(A)** LASSO coefficient profiles of the 41 baseline features, where the minimum lambda resulted in the single candidates of serum cystatin C (S-CysC) with non-zero coefficients. **(B)** Dotted vertical lines in the LASSO regression showed the optimal diagnostic model (left vertical line) and the most parsimonious model (right vertical line). The LASSO regression identifies S-CysC as the single predictor from the most parsimonious model.

In the PP set, 41 variables measured at the hospital admission (Tables 1, 2 and the kidney function variables on the first day after surgery of Table 3) were included in the LASSO regression. After the cross-validated error plot and the most parsimonious model of the LASSO regression, the S-CysC on the postoperative 1st day was identified as the single DGF predictor (Figure 2). S-CysC was the independent risk factor (β, 3.61; 95% CI, 2.53–5.15; *P* < 0.001), and the diagnostic cutoff value of the model was 3.80 mg/L (OR 10.96; 95% CI, 5.78–20.77; *P* < 0.001; Table 4). The sensitivity of PP set is 0.62 (0.48, 0.74) and the specificity is 0.87 (0.82, 0.91). The predictive effect of S-CysC on DGF preliminarily showed good discrimination with 0.797 (95% CI, 0.725–0.870; *P* < 0.001) of AUC and well-fit calibration curves, yielding approximately 50% of clinical benefit rate and predicting positives cases with 1:4 cost/benefit ratio in the PP set on the basis of 19.35% DGF incidence (Supplementary Figures 1, 2). The five times multiple imputation data of LASSO regression agreed with the S-CysC as the single DGF predictor as the most parsimonious model (Supplementary Figure 3). The postoperative 1st day S-CysC and Scr, preoperative pneumonia, and the interoperative dose of propofol between the DGF and No-DGF group showed the *P* value less than 0.1 (Supplementary Table 1). The postoperative 1st day S-CysC and preoperative pneumonia were the risk factors of DGF (β: 3.52, 95% CI: 2.43–5.10; OR: 3.45, 95%CI: 1.03–11.61, respectively; Supplementary Table 2).

**TABLE 4:** The logistic regression serum cystatin C and its Youden’s index cut-off point.

Exposure	PP set (*N* = 310) β/OR 95% CI	ITT set (*N* = 517) β/OR 95% CI
Serum cystatin C	3.61 (2.53, 5.15) **< 0.001**	3.83 (2.89, 5.08) **< 0.001**
**Serum cystatin C**		
<3.80 mg/L	1	1
≥3.80 mg/L	10.96 (5.78, 20.77) **< 0.001**	13.45 (8.02, 22.57) **< 0.001**

The P values in hold is P < 0.05.

### Predictor validation

**FIGURE 3:**
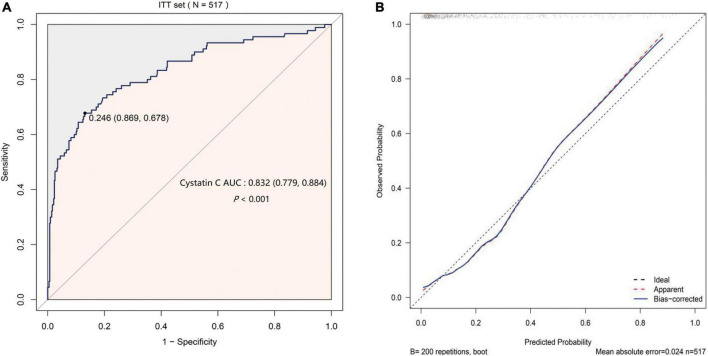
The predicted validation of serum cystatin C (S-CysC) in the Intention-To-Treat set with all values feature selection (*n* = 517). **(A)** The receiver operating characteristic curve of single S-CysC. **(B)** The calibration curve of single S-CysC on the delayed graft function (DGF) prediction. The ideal line showed the ideal estimated probabilities correspond to the actual observation; the apparent red line showed the predictive capability of the model; the bias-corrected blue line showed the predictive stability of the bootstrap corrected model. The apparent red line and the ideal dotted line had no significant different by Hosmer–Lemeshow test (*P* = 0.142), suggesting a well fit between the model and the ideal data. The apparent red line well coincided with bias-corrected blue line illustrated the stability of the prediction of S-CysC on DGF.

**FIGURE 4:**
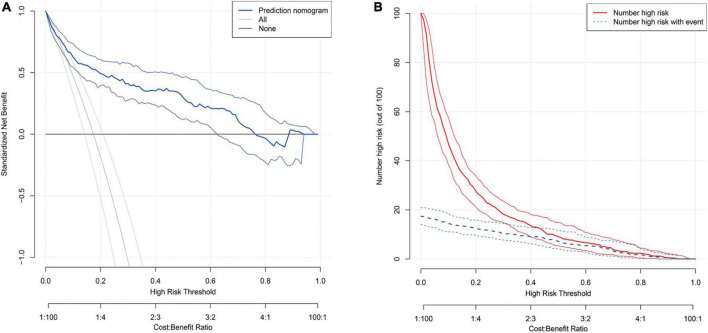
**(A)** The decision curve for the predicting delayed graft function (DGF) in the Intention-To-Treat set (*n* = 517). The thick blue line represents the model; the light gray line represents the assumption that all patients have DGF; the thick gray line represents the assumption that all patients have non-DGF. The threshold probability in the Per-Protocol set and Intention-To-Treat data set both are about 20%, using serum cystatin C (S-CysC) to diagnose DGF could yield a clinical benefit rate of 50%. **(B)** The clinical impact curve of the S-CysC based risk model showed the predicted positives cases included all the actual positives cases with 1:4 cost/benefit ratio. Of 100 patients, the heavy red solid line showed the total number who would be deemed high risk for each risk threshold. The dotted blue line shows how many of those would be true positives (cases).

The AUC of S-CysC on DGF in ITT set was 0.832 (95% CI, 0.779–0.884; *P* < 0.001), and the 200 repetitions of bootstrapping validation further confirmed this value. The calibration curve of S-CysC for the probability of DGF indicated the consistency between prediction and observation in the ITT dataset (Figure 3). The Hosmer–Lemeshow test between the apparent red line (S-CysC predictive model) and the ideal dotted line had no significant difference (*P* = 0.142), suggesting that the predictive model fitted well with the ideal model The decision curve analysis demonstrated that using this model to predict the diagnosis of DGF would have more benefits than those in all dialysis or non-dialysis patient when the threshold probability of a patient was 3–78% (Figure 4A). The incidence of DGF was 17.41% in the ITT set, and the net benefit was 50% when the model was used to make the clinical decision, compared with the -20% of net benefit in all dialysis patients and 0% of net benefit in non-dialysis patients. The clinical impact curve of the S-CysC based on the risk model showed the predicted positives cases included all the actual positives cases with 1:4 cost/benefit ratio based on the incidence of DGF (Figure 4B). Both the optimal diagnostic model and the most parsimonious model showed well predictive AUC, accuracy, sensitivity, and specificity, and had no significantly different in AUC (0.835, 95%CI 0.784–0.886 vs. 0.832, 95%CI 0.779–0.884; *P* = 0.584; Supplementary Table 3).

## Discussion

Our current retrospective study investigated the postoperative first day clinical routine renal function biomarker of S-CysC as the single predictor of DGF by the most parsimonious model of LASSO regression. The predictive cutoff value of S-CysC showed 3.80 mg/L, whose accuracy, sensitivity and specificity, respectively, were 83.6, 67.8, and 86.9%, and whose AUC had no significantly different compared with the AUC of optimal model (Supplementary Tables 1–3). The PP set, bootstrapping ITT set, and multiple imputation ITT set corresponded to the S-CysC AUCs of 0.797, 0.828, and 0.832, respectively. S-CysC showed a well-fitted calibration curve, yielding approximately 50% of clinical benefit rate and predicting positives cases with 1:4 cost/benefit ratio. The predictive effect was repeatedly validated in the ITT set with multiple interpolation data and in the data of bootstrap resamples. Our single center and retrospective study design suggested that the first postoperative S-CysC level may predict DGF.

The donor, recipient, and perioperative-related risk factors contributed to DGF incidence in 10–30% patients after kidney engraftment (23, 24). Previous predictive models focused on preoperative transplant decision, and the widely used marginal kidneys for limited donor kidney compelled clinicians to optimize postoperative clinical management decisions (25). Previous models consisted of various pretransplant items tested with simple multivariate regression and only AUC and related *P* value (7, 26). A randomized controlled trial with 78 patients reported that S-CysC combined with recipient’s and donor’s age, cold ischemia time, and urine output can predict DGF with 0.89 of AUC (18). A prospective cohort study with 40 patients reported that a formula with Scr, malondialdehyde, and S-CysC predicts DGF with 0.96 of AUC (21). However, neither of these studies proved the predictive effect of single S-CysC on DGF. Our study demonstrated the S-CysC is a single predictor of DGF with different predictor selections and verification. In addition, S-CysC as the single predictor of DGF was trained with LASSO and logistic regression from all preoperative and interoperative variables in the PP set (Figure 2). The AUC of S-CysC was 0.797 (95% CI, 0.725–0.870; *P* < 0.001). S-CysC had 50% of the net benefit of the 1:4 cost/benefit ratio based on 19.35% of the DGF incidence (Supplementary Figures 1, 2). Further, S-CysC was verified in the ITT set, in which the AUC was 0.832 (95% CI, 0.779–0.884; *P* < 0.001), and the 200 repetitions of bootstrapping validation further confirmed that the AUC was 0.828. The calibration plot diagram showed a good consistency between the actual and predicted diagnoses. The Hosmer–Lemeshow test further illustrated the predicted diagnoses, and the ideal dotted line had no significant difference (Figure 3). The kidney transplantation patients obtained a net benefit of 50% from the clinical decision of the model treatment. The net benefit had a 1:4 cost/benefit ratio based on 17.41% incidence of the DGF groups. All dialysis patients had -20% net benefits, and non-dialysis patients had 0 net benefit (Figure 4).

In this study, the Scr levels of the DGF group increased by 266.8 μmol/L relative to those of the non-DGF group (*P* < 0.001), but Scr level was not selected as the single predictor by the LASSO regression. The cohort study with 91 patients reported that Scr on postoperative first day is not predictive for AUC 0.53 (95% CI, 0.35–0.71) (27). Scr is unfit as predictor because it is derived from the balance between creatinine production and excretion, delaying the diagnosis of acute kidney injury for 48–72 h (28). Several renal tubular injury biomarkers, such as KIM-1, IL-18, and NGAL showed AUC values of 0.50 (95% CI, 0.36–0.64), 0.82 (95% CI, 0.72–0.92), and 0.82 (95% CI, 0.72–0.92) but still not available for routine use (27). S-CysC, as one of the routine renal function items, normally is reabsorbed by renal tubular epithelial cells with a low blood concentration, but it is significantly increased once the tubular is injured (29). The retrospective analysis with 47 patients showed that S-CysC, serum NGAL, and urine NGAL reflected renal function sensitively, and S-CysC reached to 4.77 mg/L with a sensitivity of 0.818 and specificity of 0.889 (30).

The strength of our study were the logical and strict predictor selection, verification, and manifestation in a large sample size of 517 patients. S-CysC was screened from all perioperative data by the most parsimonious diagnostic LASSO regression of DGF. Meanwhile, AUC, the clinical utility of the model, DCA, and clinical impact curve analysis were all implemented. Finally, the predictive effect was validated with the ITT set, bootstrap resample data, and multiple interpolation data. The postoperative first clinical routine S-CysC as a single predictor of DGF facilitates the postoperative individual patient management and hospital resources allocation in the high-risk patients with DGF. The DGF high-risk patients will be performed ultrasound examination to exclude surgery-related complications; adjusted the immunosuppressors; provided critical care or dialysis whenever need. In the future, however, the limitation of our study is a single center and retrospective study and hence its value to predict DGF warrants further prospective study. Meanwhile, it would have been more appropriate to combine S-CysC with the biomarkers of renal tubular injury, such as KIM-1, IL-18, and NGAL. Multi-biomarkers study would have helped to characterize better the complexity of DGF.

In conclusion, the postoperative first day S-CysC level may be a single predictor of DGF with good discrimination, calibration, and clinical benefit and may be used in routine clinical use, although validation studies are still needed.

## Data availability statement

The original contributions presented in this study are included in the article/Supplementary material, further inquiries can be directed to the corresponding authors.

## Ethics statement

The studies involving human participants were reviewed and approved by the First Affiliated Hospital of Xi’an Jiaotong University, Xi’an, China. Written informed consent for participation was not required for this study in accordance with the national legislation and the institutional requirements.

## Author contributions

YL and WG: study conception. YL, BW, LW, KS, WZ, SG, JC, CD, and JD: acquisition or interpretation of data. WG and YL: statistical analyses. YL, BW, and WG: drafted the manuscript. YL, BW, WG, CD, and JD: critically revised the manuscript for important intelectual content. WG: obtained funding. WG, YL, BW, LW, KS, WZ, SG, CD, and JD: provided administrative, technical, or material support. All authors contributed to the article and approved the submitted version.

## Abbreviations

AUC: area under curve
DGF: delayed graft function
DCD: donation after cardiac death
DBD: donation after brain death
DCA: decision curve analysis
ITT set: Intention-To-Treat set
IL-18: interleukin-18
KIM-1: kidney injury molecule-1
LASSO: least absolute shrinkage and selection operator
NGAL: neutrophil gelatinase-associated lipocalin
PP set: Per-Protocol set
ROC: receiver-operating characteristic curve
S-CysC: serum cystatin C
Scr: serum creatinine
β -TP: β -trace protein.
