# Supplementary material for: Postoperative day 1 serum cystatin C level predicts postoperative delayed graft function after kidney transplantation
Source: Front Med (Lausanne). 2022 Aug 12;9:863962. doi: 10.3389/fmed.2022.863962 (PMC9411520; doi:10.3389/fmed.2022.863962)
Supplement: Supplementary file 1 [file Data_Sheet_1.docx]

**Supplementary Data**


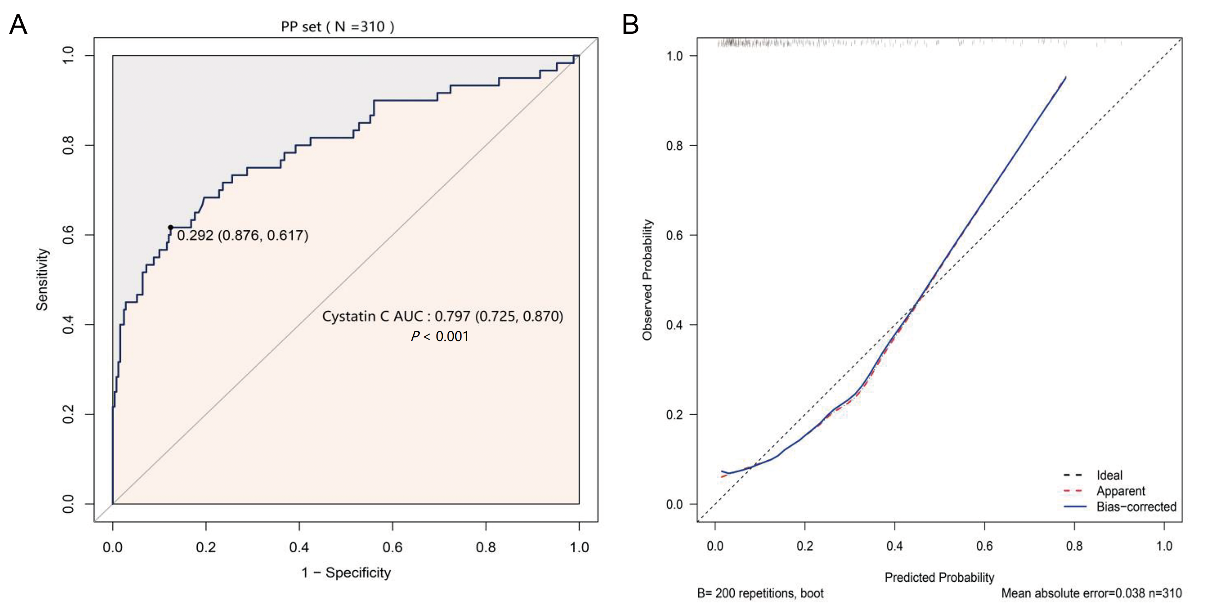


**Supplementary Figure 1** (**A)** The area under curve of the receiver-operating characteristic curve is 0.797 (95% CI: 0.725-0.870) in the Per-Protocol set (n=310). (**B)** Calibration curve in the prediction model in the Per-Protocol set (n=310). The Hosmer-Lemeshow test between the apparent red line and the ideal dotted line had no significant difference (P = 0.068).


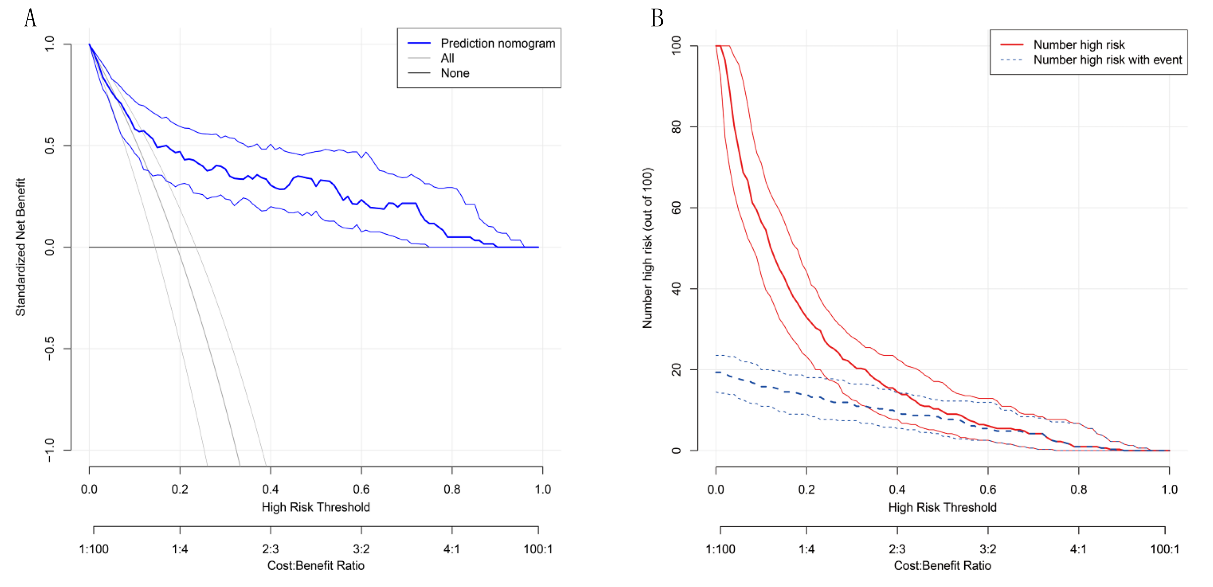


**Supplementary Figure 2 (A)** Decision curve for the predicting donation after cardiac death in renal transplant patients in the Per-Protocol set (n=310). **(B)** Clinical impact curve for predict model in the Per-Protocol set (n=310).


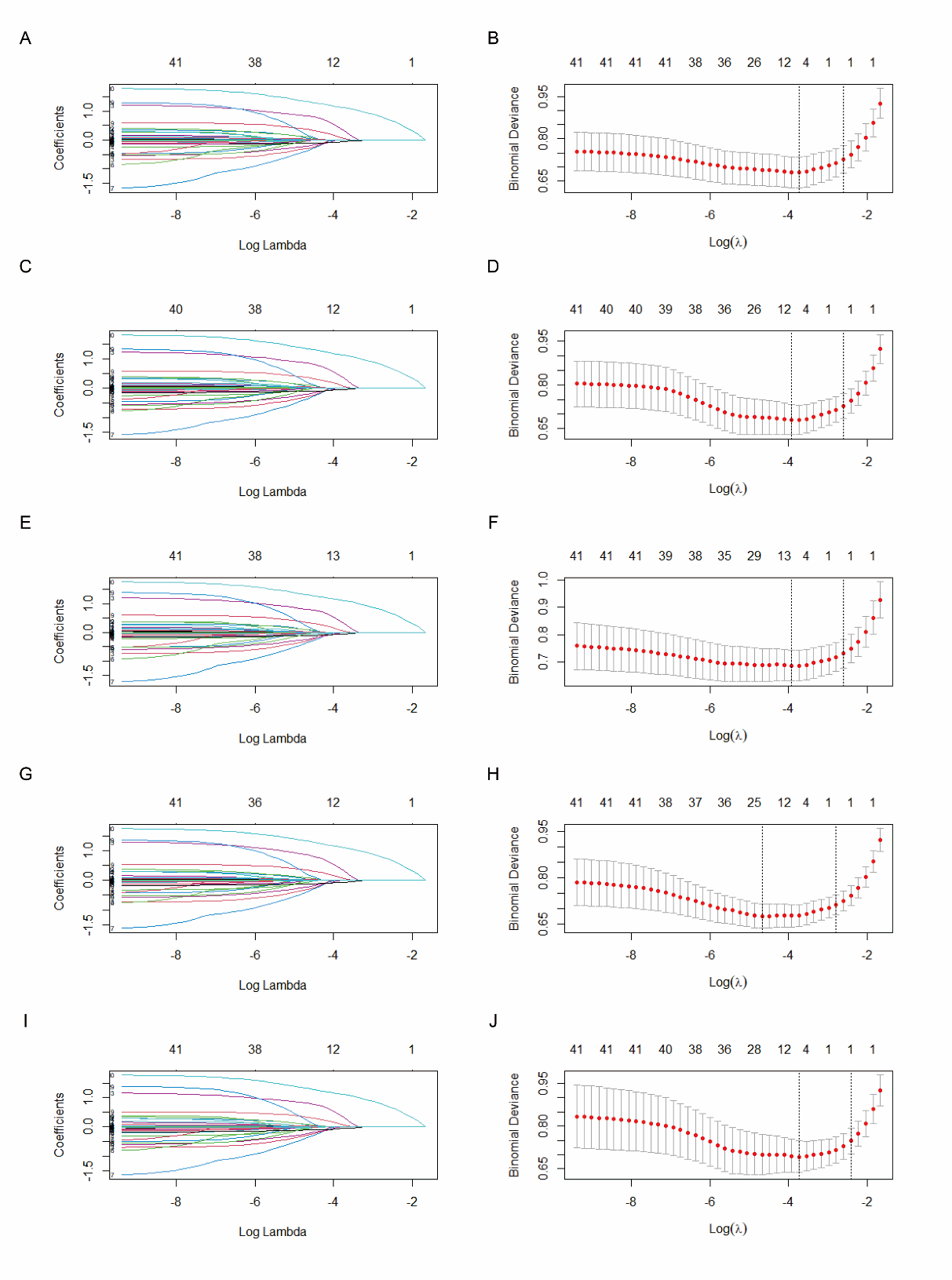


**Supplementary Figure 3** The least absolute shrinkage and selection operator binary logistic regression model in 5 times multiple interpolation of Intention-To-Treat set. The optimal diagnostic model and the most parsimonious model of LASSO regression were identified with minimum criteria and one standard error of the minimum criteria (the 1-SE criterion). The most parsimonious model (right vertical line) were the only one candidates of serum cystatin C. The least absolute shrinkage **(A)** and selection operator binary logistic regression model **(B)** in the first multiple interpolation of Intention-To-Treat set (MI-ITT1); in a similar way, **(C)** and **(D)** in MI-ITT2; **(E)** and **(F)** in MI-ITT3; **(G)** and **(H)** in MI-ITT4; **(I)** and **(J)** in MI-ITT5.

**Supplementary Table 1** P value of univariable analysis in five times multiple imputation data of optimal diagnostic model.

|  | MI-ITT1 | MI-ITT2 | MI-ITT3 | MI-ITT4 | MI-ITT5 |
| --- | --- | --- | --- | --- | --- |
| Serum Cystatin C | **<0.001** | **<0.001** | **<0.001** | **<0.001** | **<0.001** |
| Serum SCR | **<0.001** | **<0.001** | **<0.001** | **<0.001** | **<0.001** |
| Propofol, mg | **0.016** | **0.016** | **0.016** | **0.016** | **0.016** |
| Pneumonia | **0.029** | **0.029** | **0.029** | **0.029** | **0.029** |
| Colloid, ml | 0.101 |  |  | 0.101 |  |
| Hypertension | 0.178 | 0.178 | 0.178 | 0.178 | 0.178 |
| Cold Ischemia | 0.663 | 0.663 | 0.663 | 0.663 | 0.663 |
| Kidney Side (Right) | 0.912 |  |  | 0.912 |  |

**Supplementary Table 2** Multivariate analysis in five times multiple imputation data of optimal diagnostic model.

|  | OR/β(95% CI) | P-Value |
| --- | --- | --- |
| Serum Cystatin C, mg/L | 3.52(2.43,5.10) | **<0.001** |
| Pneumonia, mg | 3.45(1.03,11.61) | **0.045** |
| Propofol, mg | 1.00(1.00,1.00) | 0.157 |
| Serum SCR, μmol/L | 1.00(1.00,1.00) | 0.513 |

**Supplementary Table 3** Comparison of cystatin C as a single factor diagnosis and a multi-factor combined diagnosis of DGF.

| Test | Model 1 | Model 2 | P-Value |
| --- | --- | --- | --- |
| ROC area(AUC) 95% CI | 0.832 (0.779,0.884) | 0.835 (0.784,0.886) | 0.584 |
| Accuracy | 0.836 | 0.799 |  |
| Specificity | 0.869 | 0.810 |  |
| Sensitivity | 0.678 | 0.744 |  |

Model 1: Serum Cystatin C

Model 2: Serum Cystatin C+ Pneumonia+ Propofol+ Serum SCR
